# Retinal Ganglion Cells: Global Number, Density and Vulnerability to Glaucomatous Injury in Common Laboratory Mice

**DOI:** 10.3390/cells11172689

**Published:** 2022-08-29

**Authors:** Marie Claes, Lieve Moons

**Affiliations:** Neural Circuit Development and Regeneration Research Group, Department of Biology, KU Leuven, Leuven Brain Institute, 3000 Leuven, Belgium

**Keywords:** retinal ganglion cells, retina, glaucoma, microbead occlusion model, C57BL/6J, C57BL/6N, mice, substrains, sex-dependent differences

## Abstract

How many RBPMS^+^ retinal ganglion cells (RGCs) does a standard C57BL/6 laboratory mouse have on average and is this number substrain- or sex-dependent? Do RGCs of (European) C57BL/6J and -N mice show a different intrinsic vulnerability upon glaucomatous injury? Global RGC numbers and densities of common laboratory mice were previously determined via axon counts, retrograde tracing or BRN3A immunohistochemistry. Here, we report the global RGC number and density by exploiting the freely available tool RGCode to automatically count RGC numbers and densities on entire retinal wholemounts immunostained for the pan-RGC marker RBPMS. The intrinsic vulnerability of RGCs from different substrains to glaucomatous injury was evaluated upon introduction of the microbead occlusion model, followed by RBPMS counts, retrograde tracing and electroretinography five weeks post-injury. We demonstrate that the global RGC number and density varies between substrains, yet is not sex-dependent. C57BL/6J mice have on average 46K ± 2K RBPMS^+^ RGCs per retina, representing a global RGC density of 3268 ± 177 RGCs/mm^2^. C57BL/6N mice, on the other hand, have on average less RBPMS^+^ RGCs (41K ± 3K RGCs) and a lower density (3018 ± 189 RGCs/mm^2^). The vulnerability of the RGC population of the two C57BL/6 substrains to glaucomatous injury did, however, not differ in any of the interrogated parameters.

## 1. Introduction

Being part of the central nervous system (CNS), and alongside its accessibility, the retina is considered a highly valuable tissue to study neurodegenerative diseases. It is currently viewed as a window to the brain, allowing a non-invasive and early detection of neurodegenerative injury signs in various CNS diseases, even in those that are not primarily associated with visual system deficits, e.g., Alzheimer’s and Parkinson’s disease [1,2]. One important type of neuron that resides in the retina is the retinal ganglion cell (RGC), whose axon connects our eye to our brain. These RGCs are under attack in common CNS disorders [3], including the highly prevalent glaucoma [4], and their loss often leads to vision impairment or even blindness.

The retinal cell population of a common laboratory mouse (*Mus musculus*, C57BL/6J substrain) was first scrutinized by Jeon et al. in 1998 [5]. Historically, estimations on the RGC number in rodent species were reported using post-mortem axon counts or via retrograde tracing experiments, both exploiting the fact that the RGCs are the only afferent neurons of the retina. These methods represented the most straightforward way to assess RGC numbers before the identification of RGC markers. Nowadays—and as RGCs occupy the innermost retinal layer within the retina—RGCs can be easily assessed on (entire) wholemount retinas via immunohistochemistry with RGC markers or via murine reporter lines, both in combination with standard epifluorescence microscopy. A reporter line that specifically labels the RGC population in the murine retina is the VGLUT2-IRES-Cre × THY1-STOP-YFP mouse, as introduced by the Sanes lab [6]. These mice have been increasingly used to isolate RGCs via fluorescence-activated cell sorting (FACS) [6,7,8]. Pan-markers that specifically label RGCs include tubulin beta-3 chain (TUBB3) [9], brain-specific homeobox/POU domain protein 3A (BRN3A) [10] and RNA binding protein with multiple splicing (RBPMS) [11]. Following the identification of these RGC markers, the development of (semi) automated RGC counting algorithms on retinal wholemounts was fostered, e.g., for BRN3A [10,12,13] or RBPMS [13,14] labeling.

We recently developed a deep learning tool for the automated detection and quantification of murine RBPMS-immunopositive (RBPMS^+^) RGCs, called RGCode—short for Retinal Ganglion Cell quantification based On DEep learning [14]. Compared to manual counting on frames, fully automated counting of entire retinal wholemounts promotes scientific rigor as it allows for higher throughput, total blinding to experimental groups and reducing both bias and inter-/intra-operator variability. Additionally, it may also facilitate inter-study comparisons of RGC density data, e.g., between different mouse strains or different glaucoma models. As only a limited number of retinas was used to set up RGCode, we aimed to run a bigger pool of retinas through the tool to assess the RBPMS^+^ RGC population in common laboratory mice, i.e., C57BL/6J and -N mice. This allowed us to deduce the definitive number of RGCs in widely used laboratory mice and interrogate possible substrain- and sex-related differences in RGC counts/densities. Both substrain- and sex-related differences are important issues raised by many research groups, yet still repeatedly causing problems in the field. In addition to interrogating the global RGC count/density between C57BL/6J and -N mice, we also included a comparative analysis of retinal layer thickness via optical coherence tomography (OCT) and RGC functioning via electroretinography (positive scotopic threshold response or pSTR measurements).

The abundant and widespread use of C57BL/6J and -N mice also implies that they are bred at various locations across the globe, including vendors and independent academic colonies. This most likely introduces heterogeneity between mice from the same substrain, yet bought from a different supplier and/or bred at a different location for several generations, e.g., between European and American mice. For example, Jeon et al. reported a difference in the total number of cells in the ganglion cell layer of American versus European C57BL/6J mice [5]. In the glaucoma research field, there have been some problems with adopting the popular experimental microbead occlusion model in geographically dispersed research groups, allegedly due to differences between American versus European mice. C57BL/6N mice are known to harbor a mutation (*Rd8*) that introduces mild photoreceptor degeneration [15,16,17,18,19], possibly rendering their retinas more prone to glaucomatous injury. Mattapallil et al. reported the presence of the *Rd8* mutation in all interrogated C57BL/6N cohorts, each bought from American vendors [16], yet much less is known about European C57BL/6N mice. For this reason, we also assessed whether the RGCs of European C57BL/6J and -N mice harbor a different vulnerability to glaucomatous injury.

## 2. Materials and Methods

### 2.1. Experimental Animals

Within this study, 10–13-week-old C57BL/6J (JAX stock #000664, KU Leuven’s breeding colony, Belgium, originally acquired via Charles River Laboratories, France, the European supplier of Jax^®^ mice) or C57BL/6N (JAX stock #005304, acquired from Charles River Laboratories, Italy) mice of either sex were used and housed under standard laboratory conditions. All experiments were approved by the Institutional Ethical Committee of KU Leuven and were in accordance with the European Communities Council Directive of 22 September 2010 (2010/63/EU).

### 2.2. Glaucoma Model

The microbead occlusion model was used to induce a glaucomatous-like injury in the eyes of C57BL/6J and–N mice, according to the protocol of Ito and Belforte et al. [20] and described in more detail in [21]. Briefly, 2 µL of magnetic microbeads (Dynabeads™ M-450 Epoxy, ThermoFisher Scientific, Waltham, MA, USA) was intracamerally injected and manually repositioned with a handheld magnet towards the iridocorneal angle under general anesthesia (isoflurane, Iso-Vet 1000 mg/g, Dechra, Northwich, UK). Mice were euthanized five weeks post-microbead occlusion. 

### 2.3. Retrograde Tracing, Electroretinography and Optical Coherence Tomography

To retrogradely trace the RGCs, a foam was soaked with hydroxystilbamidine (OHSt, 4%, Life Technologies, Carlsbad, CA, USA) dissolved in saline with 10% demethylsulfoxide (Sigma-Aldrich, Saint Louis, MO, USA). Six days before euthanasia, this foam was placed on top of the superior colliculus after aspirating the overlying cortex, according to the protocol of [22] and described in more detail in [21]. For this surgical procedure, mice were sedated via an intraperitoneal mixture of medetomidine and ketamine (1 mg/kg, Domitor, Pfizer, New York City, NY, USA and 75 mg/kg, Anesketin, Eurovet, Bladel, The Netherlands), which was reversed with a subcutaneous injection of 1 mg/kg atimapezol (Antisedan, Pfizer). Functioning of RGCs was studied via the positive scotopic threshold response (pSTR), as described previously [7,21]. Briefly, mice were dark adapted overnight, one day before euthanasia. The next day, and upon pupil dilation, responses to 50 dim white light flashes (0.0001 cd·s/m^2^) were recorded in a dark room (Celeris, Diagnosys, Lowell, MA, USA) under general anesthesia (Cfr. mixture above). The amplitude was defined as the difference between the peak amplitude of the positive wave (pSTR) and the baseline signal, whereas the latency time was defined as the time between the flash onset and the occurrence of this peak amplitude of the pSTR. Next, retinal layers were imaged via spectral domain spectral domain optical coherence tomography (OCT, Envisu R2210, Bioptigen, Morrisville, NC, USA). The thickness of each layer was measured at 16 different locations across the retinal area and averaged per mouse via the InVivoVue Diver 3.0.8 software (Bioptigen), all as described previously [21].

### 2.4. Tissue Collection and RBPMS Immunohistochemistry

Mice were euthanized with an overdose of sodium pentobarbital (60 mg/kg, Dolethal, Vetoquinol, Aartselaar, Belgium), followed by transcardial perfusion with 0.9% saline and 4% paraformaldehyde (PFA), respectively. After enucleation with curved tweezers (Dumont #7 Forceps, Fine Science Tools, Heidelberg, Germany), eyes were post-fixed for 1 h in 4% PFA. Next, the eyes were 3 × 10 min submerged in phosphate buffered saline (PBS). After isolation of the retina, wholemounts were created via four radial cuts (Vannas Spring Scissors, Fine Science Tools). Hereafter, the post-fixation steps were repeated, i.e., 1 h in 4% PFA and 3 × 10 min rinsing in PBS.

For the RBPMS staining, retinas were first permeabilized by rinsing them 2 × 10 min in PBS + 0.5% Triton X-100 (ThermoFisher Scientific), followed by a freeze–thaw step at −80 °C in PBS + 0.5% Triton X-100 (15′ freeze, 30′ thaw). After two additional rinsing steps (2 × 10 min in PBS + 0.5% Triton X-100), retinas were incubated overnight with rabbit anti-RBPMS (1:250, PhosphoSolutions, Aurora, CO, USA) in a mixture of 2% blocking donkey serum (Sigma-Aldrich) and 2% Triton X-100 in PBS at room temperature. After primary antibody incubation, retinas were rinsed (3 × 10 min in PBS + 0.5% Triton X-100) and submerged in the secondary antibody mixture (Alexa-647-conjugated donkey anti-rabbit, 1:500, Life Technologies) for 2 h at room temperature. Unbound secondary antibody was washed off with PBS (3 × 10 min), and retinas were mounted with Mowiol (10%, Sigma-Aldrich). For the automated quantification of RGCs via RGCode (see below), no nuclear dyes are required. Of note, not all retinas were stained in the same batch, and some technical variability should be considered. 

### 2.5. Imaging, RGC Counting and Statistics 

Retinas were imaged with an upright, wide-field epifluorescence microscope (Leica DM6, Wetzlar, Germany). Via the Las X Navigator, each retina was outlined using a 5× objective, followed by tile scanning of the entire wholemount with a 20× objective. Imaged retinas were uploaded in the RGCode tool without any image preprocessing. RGCode is a fully automated deep learning tool that outlines the retinas and counts the RGCs, rendering information about the global RGC number and density per wholemount. RGCode was originally set up to detect RBPMS^+^ RGCs, yet as RBPMS and OHSt are both cytoplasmic labels and thus render a similar signal, RGCode was retrained to count OHSt^+^ RGCs. As such, both RBPMS^+^ and OHSt^+^ RGCs were automatically quantified via RGCode. A detailed description of this tool can be found in [14], and the tool can be downloaded via https://gitlab.com/NCDRlab/rgcode. Graphs and statistical parameters were extracted from Prism (GraphPad, San Diego, CA, USA, v9.3.1). Statistical significance was set to *p* ≤ 0.05 for all analyses, and statistical tests are provided in the figure legends. Data are reported as mean ± SD in the text and visualized as mean ± SEM in the figures. 

## 3. Results

### 3.1. Differences in Retinal Area, RGC Count and -Density between C57BL/6J and -N mice

**Figure 1:**
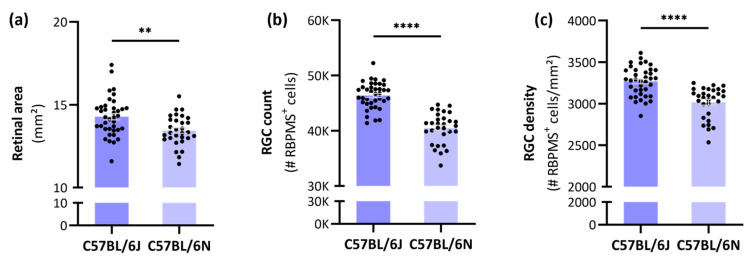
Retinal area, total RGC count and global RGC density across C57BL/6J and -N substrains. The total retinal area (**a**); RGC number (**b**); and RGC densities (**c**) of these C57BL/6 substrains followed a normal distribution (D’Agostino & Pearson test, α = 0.05). The average retinal area, RGC number and -density of C57BL/6N (*n* = 30) mice was significantly lower compared to C57BL/6J mice (*n* = 38). Unpaired, two-tailed *t*-test; ** = *p* ≤ 0.01; **** = *p* ≤ 0.0001. Automated segmentation and RBPMS^+^ RGC countings were achieved via the freely available software RGCode on entire retinal wholemounts [14].

RGCode was run on a large pool of naïve C57BL/6J mice, and the obtained parameters were compared to those of age-matched C57BL/6N mice. On average, a young adult C57BL/6J mouse has 46,395 ± 2373 RGCs in a retinal area of 14.28 ± 1.17 mm^2^, corresponding to a global RGC density of 3268 ± 177 RGCs/mm^2^ (Figure 1a–c, Table 1). Compared to C57BL/6J mice, C57BL/6N mice harbored a smaller retinal area (13.44 ± 0.91), lower RGC count (40,501 ± 2788 RGCs) and lower RGC density (3018 ± 189 RGCs/mm^2^).

### 3.2. No Sex-Related Differences in Retinal Area, Global RGC Number or Density

**Figure 2:**
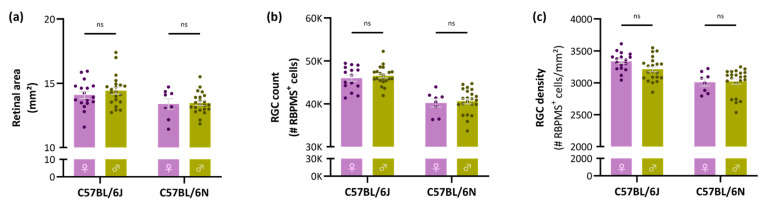
Retinal area, total RGC count and global RGC density per sex in C57BL/6J and -N mice. Retinal area (**a**); RGC number (**b**); and density (**c**) did not differ between female (*n* = 17 for C57BL/6J, 8 for C57BL/6N) and male (*n* = 21 for C57BL/6J, 22 for C57BL/6N) mice and is thus not sex-dependent. Two-way ANOVA with Tukey’s post hoc test; ns = non-significant.

To evaluate whether the differences between C57BL/6J and -N mice could be sex-dependent, the data were split according to their sex. Notably, no differences in retinal area, RGC count or RGC density between female versus male mice were observed for any of the studied substrains (Figure 2a–c, Table 1).

### 3.3. Mild Photoreceptor Layer Thinning in C57BL/6N Mice but No Difference in RGC Functioning between C57BL/6 Substrains

**Figure 3:**
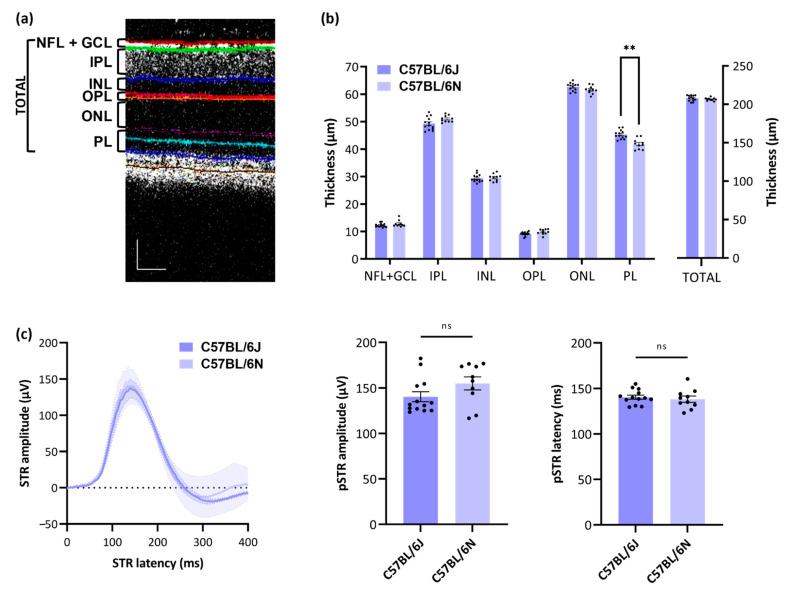
Retinal layer thickness and RGC function in C57BL/6J versus –N mice: (**a**) overview of different retinal layers acquired via optical coherence tomography (OCT); scale bar = 50 µm; (**b**) mild thinning of the photoreceptor layer is apparent in C57BL/6N (*n* = 10) versus C57BL/6J (*n* = 13) mice. Two-way ANOVA with Šídák’s post hoc test, ** *p* ≤ 0.01; (**c**) no difference in pSTR amplitude or latency was detected, revealing identical functioning of RGCs in C57BL/6J (*n* = 13) versus C57BL/6N (*n* = 10) mice; unpaired, two-tailed *t*-test, ns = non-significant. Key: NFL = nerve fiber layer; GCL = ganglion cell layer; IPL = inner plexiform layer; INL = inner nuclear layer; OPL = outer plexiform layer; ONL = outer nuclear layer; PL = photoreceptor layer; TOTAL = total neuroretina.

To study the differences in RGC count and density in more depth, the thickness of the retinal layers was studied via OCT (Figure 3a,b), and RGC functioning was interrogated via pSTR measurements (Figure 3c). Modest retinal layer thinning was observed in the photoreceptor layer of C57BL/6N mice compared to C57BL/6J mice, corresponding to a thinning of 7.60 ± 4.22%. The thickness of other retinal layers as well as the total neuroretina did not differ between C57BL/6J and -N mice (Figure 3b, Table 2). RGC functioning was also not found different between the two substrains, both in terms of pSTR amplitude and latency time (Figure 3c).

### 3.4. No Substrain-Dependent Differences in RGC Vulnerability to Glaucomatous Damage

**Figure 4:**
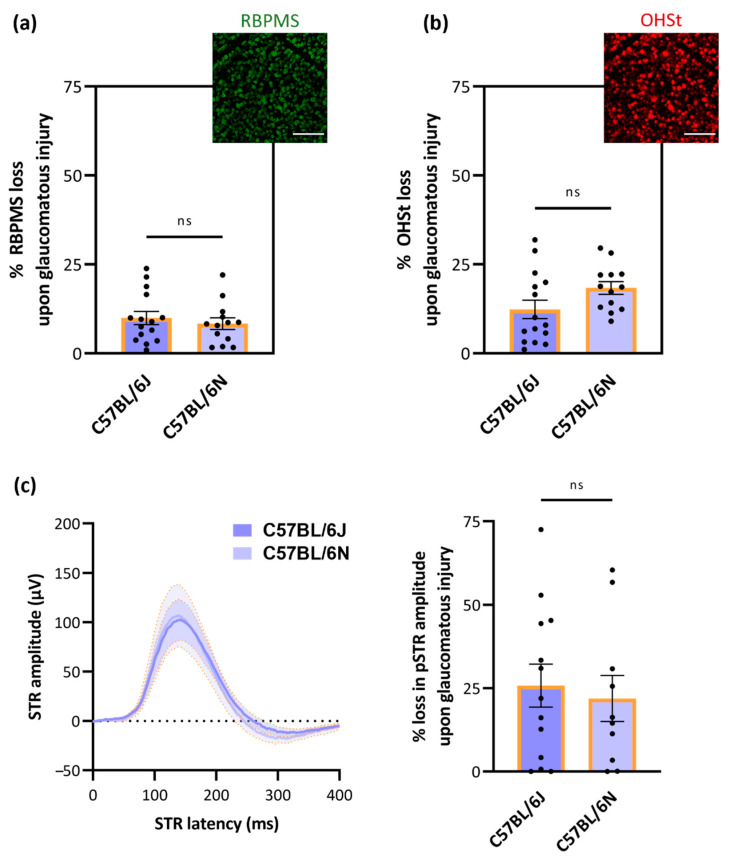
Loss of RBPMS^+^ and OHSt^+^ RGCs versus decline of RGC functionality across (young) C57BL/6J and -N mice with glaucomatous, microbead-occluded eyes. At five weeks post-microbead injection, no difference in RGC susceptibility was observed: the loss of RBPMS^+^ RGCs (**a**) and OHSt^+^ RGCs (**b**)—both quantified via the automatic counting tool RGCode—was identical in each substrain (*n* = 15 for C57BL/6J mice and 13 for C57BL/6N mice). Similarly, no difference in the decline of pSTR amplitude (**c**) upon glaucomatous injury was observed between C57BL/6J- and N mice (*n* = 13 for C57BL/6J mice and 10 for C57BL/6N mice). RBPMS, OHSt and pSTR amplitude loss is expressed relative (%) to the values of the contralateral uninjured eyes. Scale bar = 100 µm. Unpaired, two-tailed *t*-test (% RBPMS and OHSt loss) or one-ANOVA with Tukey’s post hoc test (loss in pSTR amplitude); ns = non-significant.

In addition to strain- and sex-dependent differences in RGC number/density, we evaluated strain-dependent differences in the susceptibility of RGCs to glaucomatous injury. For this purpose, the most widely employed experimental glaucoma model, i.e., the microbead occlusion model, was used. The effect of glaucomatous injury on RGC numbers was evaluated via RBPMS labeling (Figure 4a), retrograde tracing with OHSt (Figure 4b) and pSTR measurements (Figure 4c), all five weeks after the induction of the glaucomatous pathology. No difference in the susceptibility of the RGC population was noted between C57BL/6J and -N mice in any of the studied parameters (Figure 4a–c). Mean loss of RBMPS^+^ RGCs in C57BL/6J was on average 9.94 ± 7.10% versus 8.35 ± 5.93% in C57BL/6N mice. The average loss of OHSt^+^ RGCs was estimated at 12.36 ± 10.02% and 18.38 ± 6.36% RGCs in C57BL/6J and -N mice, respectively. Last, C57BL/6J and -N mice also showed a similar decline in pSTR amplitude: 25.79 ± 23.15% versus 21.93 ± 21.84%, respectively.

## 4. Discussion

### 4.1. The Total Number of RGCs in C57BL/6J and -N Mice

The total number of RGCs in standard laboratory mice is reckoned to range between 40,000 and 60,000 cells, representing ± 1% of the total retinal cells. With the help of our freely available deep learning model to count RGCs on RBMPS-stained wholemounts—RGCode [14]—we studied differences in retinal area, global RGC count and -density in two C57BL/6 substrains, i.e., C57BL/6J and -N. The average RGC numbers and standard deviations reported here as assessed via the pan-RGC marker RBPMS highly correspond to other studies, in which the RGC number was determined via axon counts, retrograde tracing or BRN3A immunolabeling [5,12,23,24,25,26,27,28,29,30,31] (Table 3). Similarly, the total retinal area obtained in our study is similar to previous observations, who reported an average retinal area of 14.6 ± 0.9 mm^2^ in C57BL/6 mice [23]. The computed global densities of naive C57BL/6J and -N retinas are also in line with previous reports. RGC densities in C57BL/6 mice are usually estimated to be around 3000 RGCs/mm^2^, calculated after retrograde tracings [23,26,32] or with manual counts of RGCs on wholemount retinas after RBPMS immunolabeling [33,34]. 

**Table 3:** Global RGC counts in C57BL/6 mice assessed via axonal counts, retrograde tracing or BRN3A immunolabeling. Key: - = not reported.

RGC Labeling Method	Mean Number of RGCs ± SD	C57BL/6 Substrain	Sex	Sample Size	References
**Axon counts**	54,630 ± 3910	J	Mixed	21	[24]
	44,857 ± 3125	J	-	4	[5]
	46,000 ± 1000	-	Male	-	[25]
	51,064 ± 5045	-	Female	97	[27]
	44,846 ± 3980	J	Mixed	7	[28]
	41,659 ± 2700	J	Male	10	[30]
**Retrograde tracing from optic nerve**	50,920 ± 1161	-	Mixed	5	[26]
	49,823 ± 1792	J	Mixed	9	[31]
	42,658 ± 1540	N	Male	10	[23]
**Retrograde tracing from target area**	41,192 ± 3395	N	Male	42	[23]
	40,437 ± 3196	N	Female	9	[35]
**BRN3A counts ** **on entire wholemounts**	34,627 ± 1821	N	Female	9	[35]
	45,637 ± 2632	J	Mixed	8	[12]
**RBPMS counts ** **on entire wholemounts**	46,395 ± 2373	J	Mixed	38	Current study
	40,501 ± 2788	N	Mixed	30	Current study

### 4.2. Substrain-Dependent Differences in Retinal Area, RGC Count and -Density

Nowadays, RGC numbers/densities in murine models are estimated via RGCs somas counts on retinal wholemounts instead of axonal counts on optic nerve cross sections (Cfr. Introduction). However, most research groups still manually count RGCs on retinal sections or on small sampling areas from retinal wholemounts—not covering the entirety of the retina. The global RGC number/density is then approximated via area calculations, which are rough estimates as the RGC density greatly varies between the central and peripheral retina. Our automated approach, i.e., the deep learning tool RGCode, enables the quantification of entire retinal wholemounts, providing a precise assessment of the entire RGC population. In addition to these differences in the employed technique to assess the RGC number, variations in RGC number/density estimates could be explained by (sub)strain differences. Even between cohorts of identical (inbred) mouse strain, differences are denoted, especially when bought from different suppliers and thus bred at different locations, as evidenced by [5,24]. C57BL/6J and -N mice are, by far, the most commonly used inbred laboratory mice in neuroscience. While originally derived from the same parental mice, comparative genome sequencing has identified apparent genetic differences between C57BL/6 substrains [36,37,38,39,40]. In ophthalmologic research, the use of C57BL/6J mice is preferred over C57BL/6N as the latter harbor a universally spread *Rd8* mutation in the *Crb1* gene across all C57BL/6N mice. This mutation is associated with mild photoreceptor disorganization and degeneration, which worsens upon aging [15,16,17,18,19]. Reported ophthalmologic dissimilarities between both substrains include differences in retinal organization, visual acuity (optomotor response) [38], number of retinal vessels, occurrence of white spots (fundus endoscopy) [15,16,38,41], response to circadian disruption [42] and expression of pro-inflammatory markers [43,44]. 

Notably, both the retinal area and RGC number were found significantly lower in C57BL/6N mice compared to C57BL/6J mice in our study. The lower RGC number was, however, not proportional to the smaller retinal area, as the total RGC density was also significantly lower in C57BL/6N mice. The reported differences in global RGC count are in accordance with the study of Williams et al. in 1996, who compared the RGC axon number of different inbred and outbred laboratory mouse strains [24]. The authors did not compare C57BL/6J and -N mice, but they did show a remarkable difference (±17%) in RGC number between C57BL/6J cohorts originating from two different Jackson Laboratory colonies (Bar Harbor, ME, USA). Of note, various reports mention the use of “C57BL/6” mice but do not always specify the substrain and/or breeder (Table 3). As evidenced by our findings and by others, genetic background effects could be a confounding factor in any study. Hence, we urge breeders as well as scientists to thoroughly document any information regarding the experimental mice to guarantee reliability and reproducibility of the research data.

### 4.3. No Sex-Related Differences in Retinal Area, Global RGC Number or Density

Gender differences are widely known to affect disease prevalence and accompanying treatments, including the well-known and persisting gender bias in clinical research [45]. In glaucoma, gender is an acknowledged risk factor, with a higher incidence in women [46,47]. Despite all this knowledge, many animal studies use mixed-sex cohorts and little attention—especially in the field of glaucoma—has been paid to how male and female mice respond differently in preclinical studies. In humans, gender-related differences were found both on a structural (OCT of retinal layers) [48,49] and functional (electroretinography) [50,51] level. Sex-related differences in the visual system of mice are also noted, including differences in contrast sensitivity [52], divergent age-related changes in retinal gene expression [53] and accelerated degeneration in female retinal degeneration models [54,55,56]. In C57BL/6N mice, *Rd8* lesions are also more common in male versus female mice [15]. 

In our study, the difference in RGC count and density between C57BL/6J and -N mice could, however, not be explained by sexual dimorphism, as no significant differences between the retinal area, global RGC count or density between female and male mice were found. This finding is also in accordance to the Williams study, who also did not detect sex differences in RGC number [24]. Hence, mixed-sex cohorts of C57BL/6J or -N mice can be used in the study of RGC number/density, yet one should always bear in mind that responses to any injury model and/or therapy could differ in male versus female mice in such preclinical studies. 

### 4.4. Mild Photoreceptor Layer Thinning in C57BL/6N Mice but No Difference in RGC Functioning between C57BL/6 Substrains

Building on the finding that the RGC density differs between C57BL/6 substrains, we evaluated retinal layer thickness via OCT and RGC functioning via pSTR measurements. In line with reports showing identical retina-wide functioning via full-field flash electroretinography between wildtype and *Rd8* mice [19,57], we did not observe a difference in RGC functioning between both substrains. The global thickness of the (neuro)retina was unaltered, while thinning of the photoreceptor layer was apparent in C57BL/6N mice. This thinning could probably be attributed to *Rd8* mutation that primarily affects the photoreceptors in C57BL/6N mice [15], although we did not verify the presence of the *Rd8* mutation in our mouse cohort Our reported values for the thickness of each retinal layer in C57BL/6J and- N mice via OCT imaging highly correspond to those reported by Moore et al. [15] and Ferguson et al. [58], respectively.

### 4.5. No Substrain-Dependent Differences in RGC Vulnerability to Glaucomatous Damage

We previously showed a difference in RGC susceptibility to glaucomatous damage in pigmented (C57BL/6N) versus albino (CD-1) mice [59]. However, in the same study, we did not observe a difference between wildtype and genetically modified C57BL/6N mice, the latter being albino C57BL/6N-Tyr^C^ mice with a single homozygous Cys103Ser mutation. In the current study, we evaluated the intrinsic vulnerability of RGCs to glaucomatous damage upon microbead occlusion in two commonly used laboratory mouse strains, i.e., C57BL/6J and -N mice. Interestingly, and although the C57BL/6N mice possess a mutation that is associated with retinal degeneration, no differences in RGC loss, axonal transport loss or loss of RGC functioning were observed. This finding is in accordance with other studies reporting no difference in susceptibility of C57BL/6J and -N mice to retinal damage, e.g., after autoimmune optic neuritis [19], laser-induced choroidal neovascularization [44] or light-induced apoptosis [60]. Of note, all parameters were evaluated at five weeks post-microbead occlusion, as significant RGC loss was detected from this sampling time point on. Proportional to the average reduction of RBPMS^+^ cells, the loss of OHSt^+^ cells was slightly higher. This marked difference denotes the percentage of RGCs that are disconnected from their target area, yet still alive, and/or the occurrence of retrograde transport losses in the microbead occlusion model. However, comparing structural with functional RGC loss revealed that functional deficits precede structural ones. The decline in pSTR peak amplitude was proportionally more than twice as large as the reduction in RBPMS^+^ cell number. Hence, the pSTR seems to be a more sensitive measure to evaluate the effect of mild ocular hypertension on RGCs as compared to RBPMS immunolabeling, as also discussed in [21].

A last discussion point we would like to briefly highlight is the occurrence of a contralateral effect after a unilateral injury, also referred to as the mirror effect. Various reports denote responses in the contralateral, uninjured eye after unilateral optic nerve injury, including molecular changes, neuroinflammation and even cell death, often proportional to the severity of the retinal insult [61,62,63,64,65,66,67,68]. In addition, in pressure-dependent glaucoma models, a bilateral glial response has been previously denoted upon unilateral injury, e.g., in the episcleral vein cauterization model [69], laser photocoagulation model [70] and the microbead occlusion model [71]. In the microbead occlusion model, the Calkins lab reported a redistribution of astrocyte-derived metabolites from unstressed (contralateral) to stressed (microbead occluded) optic nerves [71]. In our study, however, we did not observe anatomical (RBPMS density) or functional (pSTR) differences between naïve and contralateral eyes five weeks after unilateral microbead occlusion (data not shown).

## 5. Conclusions

In the search towards neuroprotective strategies, the quantification of RGC numbers offers a measurable end point to determine the degree of protection. In that context, knowing the total RGC number and/or global densities in standard laboratory animals is a prerequisite, alongside the use of proper control mice. In this report, we documented the global, normative RGC numbers/densities of two most commonly used laboratory mice in neuroscience, i.e., C57BL/6J and -N mice of ± 3 months old, by automated countings of RBPMS^+^ cells on entire wholemount retinas via the deep learning tool RGCode. We highlighted differences in RGC numbers/densities between (European) C57BL/6J and -N mice. Once more, this study provides a valuable warning to the vision science community to be mindful when choosing control mice, i.e., using controls with an identical genetic background and preferably even littermates, as well as to provide detailed descriptions of the experimental mice in any research communication. Although we did not detect sexual dimorphism in RGC number/density, nor any substrain-dependent differences in RGC vulnerability to glaucomatous damage, we wish to advise researchers to always validate whether sex or genetic differences are a cofounding factor in their study.

## Figures and Tables

**Table 1:** Overview of retinal area, global RGC count and -density for C57BL/6J and -N mice, per sex and in total (sex-mixed). Values were calculated using the automated tool RGCode and are reported as mean ± SD.

	C57BL/6J		C57BL/6N	
	Female	Male	Female	Male
**Sample size**	17	21	8	22
**Area** (mm^2^)	14.09 ± 1.11	14.43 ± 1.22	13.4 ± 1.16	13.46 ± 0.84
	14.28 ± 1.17		13.44 ± 0.91	
**Count** (number of RBPMS^+^ cells)	46,225 ± 2655	46,533 ± 2178	40,203 ± 2685	40,609 ± 2880
	46,395 ± 2,373		40,501 ± 2788	
**Density** (number of RBPMS^+^ cells/mm^2^)	3336 ± 144	3212 ± 184	3009 ± 161	3021 ± 201
	3268 ± 177		3018 ± 189	

**Table 2:** Overview of average retinal layer thickness in C57BL/6J and -N mice, measured via OCT imaging. Data are shown as mean ± SD. Key: NFL = nerve fiber layer; GCL = ganglion cell layer; IPL = inner plexiform layer; INL = inner nuclear layer; OPL = outer plexiform layer; ONL = outer nuclear layer; PL = photoreceptor layer; TOTAL = total neuroretina.

	Retinal Layer Thickness (µm)						
	NFL + GCL	IPL	INL	OPL	ONL	PL	TOTAL
**C75BL/6J** (*n* = 13)	12.27 ± 0.70	49.25 ± 2.36	29.36 ± 1.40	9.11 ± 0.70	62.68 ± 1.44	45.30 ± 1.60	208.00 ± 3.63
**C75BL/6N** (*n* = 10)	12.80 ± 1.18	50.99 ± 1.20	29.59 ± 1.32	9.85 ± 0.94	61.58 ± 1.44	41.86 ± 1.91	206.60 ± 1.90

## Data Availability

Not applicable.
